# Mediterranean Diet and Diabetes: Prevention and Treatment

**DOI:** 10.3390/nu6041406

**Published:** 2014-04-04

**Authors:** Michael Georgoulis, Meropi D. Kontogianni, Nikos Yiannakouris

**Affiliations:** Department of Nutrition and Dietetics, Harokopio University, 70 El. Venizelou Street, Athens 17671, Greece; E-Mails: mihalis.georgoulis@gmail.com (M.G.); mkont@hua.gr (M.D.K.)

**Keywords:** Mediterranean diet, dietary patterns, diabetes, glycemic control, obesity, cardiovascular diseases, prevention, treatment, public health

## Abstract

The aim of the present review is to examine current scientific knowledge on the association between the Mediterranean diet and diabetes mellitus (mostly type 2 diabetes). A definition of the Mediterranean diet and the tools widely used to evaluate adherence to this traditional diet (Mediterranean diet indices) are briefly presented. The review focuses on epidemiological data linking adherence to the Mediterranean diet with the risk of diabetes development, as well as evidence from interventional studies assessing the effect of the Mediterranean diet on diabetes control and the management of diabetes-related complications. The above mentioned data are explored on the basis of evaluating the Mediterranean diet as a whole dietary pattern, rather than focusing on the effect of its individual components. Possible protective mechanisms of the Mediterranean diet against diabetes are also briefly discussed.

## 1. Introduction

Diabetes mellitus (DM) is a group of metabolic diseases characterized by hyperglycemia, resulting from defects in insulin secretion, insulin action, or both. Long considered a disease of minor significance, in the 21st century, DM represents one of the main threats to human health. In 2010, an estimated 285 million individuals worldwide are suffering from DM, with type 2 diabetes mellitus (T2DM) accounting for most cases [1]. Although it is well established that both genetic and environmental factors contribute to the development and progression of T2DM, the recent dramatic increase in its prevalence seems to result mainly from the major lifestyle changes observed in modern societies [2]. Fortunately, lifestyle factors, including dietary and physical activity habits, are largely modifiable and are currently targeted as a mean to prevent and treat the disease.

With regard to dietary habits, current trends in nutritional epidemiology propose that pattern analysis is the most realistic approach to examine associations between overall diet and health or disease, instead of focusing on single dietary components [3]. Indeed, several dietary patterns have been proven beneficial both for the prevention and management of T2DM; most of these dietary patterns are characterised by high consumption of plant foods and low consumption of animal-based, high-fat and processed foods [4]. The Mediterranean diet (MD) is a primarily plant-based dietary pattern, that has long been celebrated for its various health benefits, mainly in relation to decreased risk of cardiovascular diseases (CVD) and cancer, as well as decreased all-cause and disease-specific mortality [5]. Interestingly, mounting evidence indicates a favourable effect of the MD on T2DM as well.

The aim of the present review is to provide an overview of the current literature exploring the role of the MD in DM (mainly T2DM) prevention and treatment, as well as to discuss potential mechanisms by which MD protects individuals against the disease. In this narrative review, data from epidemiological and interventional studies published in the Medline (PubMed) database evaluating the association between the MD and DM in humans were included. Emphasis was given on reports assessing the MD as a whole dietary pattern, rather than its individual components (e.g., olive oil consumption, vegetables consumption *etc*.). Key words used in the search process included Mediterranean diet and diabetes, insulin resistance, glycemic control, glucose homeostasis, diabetes complications, cardiovascular diseases, cardiovascular mortality, as well as combinations of these.

## 2. Definition and Assessment of Adherence to the Mediterranean Diet

The term MD was originally conceived by Ancel Keys in the Seven Countries Study [6], based on his observation of the dietary habits of some populations in the Mediterranean region. These unique dietary habits were thought to be the main reason for the strikingly low rates of cardiovascular and neoplastic diseases observed in the Mediterranean communities (Italy and Greece), compared with the other studied populations of the Seven Countries’ Study [6]. The MD can be described as the dietary pattern adopted in the olive growing areas of the Mediterranean region in the late 50s and early 60s, when the region was recovering from the effects of World War II but was not yet influenced by the rising trend of fast food [7]. It is noteworthy that there is no single MD; different regions in the Mediterranean basin have their own diets, varying in several parameters, but also presenting many common characteristics. For example, total daily lipid intake may be high as in Greece (~40% of total energy intake), or moderate as in Italy (~30% of total energy intake), however in both cases the main source of dietary lipids is monounsaturated fat [7].

In addition, although the MD is a dietary pattern with plenty, well-established health benefits, a progressive shift towards westernised dietary habits has lately occurred in the Mediterranean regions [8,9]. As a result, the term “Mediterranean-style diet” is currently used in the literature in order to describe not a specific diet, but rather a collection of dietary habits traditionally followed by the populations of countries bordering the Mediterranean Sea [10]. In brief, a Mediterranean-style diet is characterised by high consumption of olive oil (as the main edible fat), vegetables, legumes, whole grains, fruits and nuts, moderate consumption of poultry and fish (varying with proximity to the sea), low consumption of full fat dairy products and red meat, and low-to-moderate consumption of wine as the main source of alcohol accompanying meals [10].

For several decades, the dominant approach used to test the association between diet and health indices was based on exploring the effects of single nutrients, foods or food groups. However, the fact that individuals consume complex combinations of foods consisting of several nutrients that interact with each other, led to the emergence of the dietary pattern analysis, aiming to explore the association between overall diet and health or disease [11]. Two fundamental indirect approaches have been so far applied for the estimation of adherence to dietary patterns; the “theoretically defined dietary patterns” approach (a-priori analysis), which involves the composition of predefined dietary quality indices based on current nutritional knowledge (*i.e*., specific dietary guidelines or diets known to be healthy) and the “empirical adherence to dietary patterns” approach (a-posteriori analysis), which is based on statistical methods (e.g., principal component analysis, cluster analysis *etc*.) in order to explore the dietary patterns adopted by the study population [12,13]. The vast majority of the scores for the estimation of adherence to the MD have been constructed based on the first approach. MD indices are combined measures of individual components (usually numeric expressions of food, food group or combination of nutrient, food and food group intakes), which are scored using specific cut off points and then summed in order to develop a total score. So far, several a-priori MD scores have been proposed, varying in the number and sort of components included as well as the components’ scoring system, with higher values in these scores reflecting higher adherence to the MD (for a review see 13). Although methodological issues regarding their composition and their diagnostic ability remain unresolved, MD indices have been widely used in research to explore the association between adherence to the MD and risk of chronic diseases [13].

## 3. Mediterranean Diet and Risk of Diabetes

Given the life-threatening complications of DM that can lead to severe disability or premature death, strategies aiming to prevent the disease are of major public health importance. With regard to long-term dietary habits, several “healthy” dietary patterns have been inversely associated with the risk of DM development [14]. These protective dietary patterns include both a-priori defined patterns, such as the DASH (Dietary Approach to Stop Hypertension) diet or dietary patterns assessed by the AHEI (Alternative Healthy Eating Index) and the GFPI (German Food Pyramid Index) indexes and various a-posteriori defined prudent/healthy dietary patterns, derived by factor or cluster analysis. Despite variation among studies, most of these protective dietary patterns present many similarities with the traditional MD, since they are mostly plant-based and include a high consumption of whole-grain foods, fruits and vegetables. Indeed, the link between adherence to the MD and DM prevention has been so far explored in several studies, with the vast majority of the data supporting a beneficial effect of the MD, as described below.

Cross-sectional evidence regarding the association between adherence to the MD and the likelihood of DM is limited. Regarding T2DM, data are available from two large population-based studies; the ATTICA study, carried out in 1514 men and 1528 women living in the province of Attica Greece, in which adherence to the MD was inversely associated with the odds of T2DM [15] and the Di@bet.es study, a national survey aiming at examining the prevalence of impaired glucose regulation in 5076 individuals from Spain, which failed to report similar results [16]. Besides T2DM, a recent multicentre observational study among 1076 pregnant women from ten Mediterranean countries also supports an inverse association between adherence to the MD and the likelihood of gestational DM [17]. It should be noted, that although cross-sectional studies provide some insight into the association between adherence to the MD and DM, they also present many methodological limitations, mainly due to the uncertainty regarding the temporality of the association. For instance, it is not clear whether the observed dietary habits precede the development of DM or reflect dietary modifications made by patients after the diagnosis of the disease. Hence, a cross-sectional design does not allow the reporting of a causal link between adherence to the MD and DM risk.

More consistent and enlightening are the results of studies with prospective design providing estimates of DM risk according to different levels of adherence to the MD [18,19,20,21,22,23,24,25], which are briefly presented in Table 1. All available studies [18,19,20,21,22,23,24,25] support the protective role of the MD against T2DM development with overall reductions in risk ranging from 12% to 83% for subjects closely adhering to the MD, compared to those reporting the lowest adherence, after adjusting for several confounding factors (e.g., sex, age, smoking, family history of DM, body mass index, physical activity levels, daily energy intake, *etc*.). All studies provide estimates of T2DM risk except one study that specifically assessed the association between pre-pregnancy adherence to the MD and risk of gestational DM [22]. Interestingly, the MD has been shown to protect against T2DM not only in healthy individuals [19,20,21,25], but also in women with a history of gestational DM [23] and individuals with established CVD [18].

Randomized controlled trials (RCT) are considered the gold standard for clinical trials when testing the effectiveness or efficacy of an intervention and for reporting causal inference. Apart from the aforementioned cohort studies, there is also a RCT, which so far provides the strongest evidence on the association between MD and T2DM development [26]. In this interventional study (PREDIMED study), 418 non-diabetic high cardiovascular risk subjects were randomly assigned to education on either a low-fat diet or to one of two MDs, supplemented with either free virgin olive oil (1 L per week) or nuts (30 g per day). After a mean follow-up of 4 years, participants allocated in the MD supplemented with olive oil and nuts groups exhibited a 51% (95% confidence interval (CI) 0.25–0.97) and 52% (95% CI 0.24–0.96) reduced risk of T2DM, respectively, compared with the low-fat diet group, after adjusting for several confounders (*i.e*., age, sex, baseline energy intake, body mass index, waist circumference, physical activity, smoking status, fasting serum glucose, use of lipid-lowering drugs, Mediterranean diet score and weight changes during the study). When the two MD groups were pooled and compared with the control group, diabetes incidence was reduced by 52% (95% CI 0.27–0.86).

**Table 1:** Studies with prospective design exploring the association between adherence to the Mediterranean diet and risk of diabetes mellitus.

Reference	Study Sample, Design and Methods	Results
Mozaffarian *et al*. [18]	Prospective study with a sample of 8291 Italian patients with a recent myocardial infarction participating in the GISSI-Prevenzione trial, who were free of T2DM at baseline and followed up for a median of 3.5 years. Adherence to the MD was assessed using a score incorporating a few typical components of the traditional MD *.	Participants in the highest quintile of adherence to the MD (MD score > 10) exhibited a 35% lower risk of T2DM (95% CI 0.49–0.85), compared with those in the lowest quintile (MD score < 6). When individual components of the score were evaluated, only consumption of cooked vegetables was significantly associated with T2DM risk (HR = 0.65, 95% CI 0.43–0.99).
Martinez-Gonzalez *et al*. [19]	Prospective cohort study with a sample of 13,380 Spanish university graduates from the SUN cohort study, who were free of T2DM at baseline and followed up for a median of 4.4 years. Adherence to the MD was assessed with the Mediterranean Diet Scale (MDS) score ^§^.	Participants with high adherence to the MD (MDS > 6) exhibited a 83% (95% CI 0.04–0.72) reduced risk of T2DM, compared with those with low adherence (MDS < 3). A 2-unit increase in the MDS score was associated with a 35% (95% CI 0.44–0.95) reduced risk of T2DM.
de Koning *et al*. [20]	Prospective cohort study with a sample of 41,615 initially free of T2DM, CVD or cancer men from the Health Professionals Follow-Up Study who were followed up for ≤20 years. Adherence to the MD was assessed with the alternate Mediterranean Diet (aMED) score ^†^.	Participants in the highest quintile of adherence to the MD (aMED > 6) exhibited a 25% (95% CI 0.66–0.86) decreased risk of T2DM, compared with those in the lowest quintile (aMED < 3); risk reduction was greater among overweight or obese subjects, compared with normal-weight ones (*p* < 0.01).
Romaguera *et al*. [21]	Case-cohort study with a sample of 11,994 incident T2DM case subjects and a stratified subcohort of 15,798 participants with 3.99 million person-years of follow-up selected from the total cohort of the European Prospective Investigation into Cancer and Nutrition (EPIC) study. Adherence to the MD was assessed using the relative Mediterranean diet (rMED) score ^‡^.	Participants with high adherence to the MD (rMED > 10) exhibited a 12% (95% CI 0.79–0.97) reduced risk of T2DM, compared with those with low adherence (rMED < 7); these results were attenuated when alcohol, meat, and olive oil components were excluded from the score, suggesting their significant contribution to the aforementioned association.
Tobias *et al*. [22]	Prospective cohort study with a sample of 15,254 healthy women from the Nurses’ Health Study II (NHS II), reporting 21,376 singleton live births from 1991 to 2001. Adherence to the MD was assessed using the aMED score and a modified version of the aMED score^ †^.	Women in the highest quartile of adherence to the MD (mean aMED = 6.6 ± 0.7) exhibited a 24% (95% CI 0.60–0.95) reduced risk of gestational DM, compared with those in the lowest quartile (mean aMED = 1.6 ± 0.6). Results were similar for the modified aMED score (HR = 0.75, 95% CI 0.61–0.91).
Tobias *et al*. [23]	Prospective cohort study with a sample of 4423 women from the Nurses’ Health Study II (NHS II) with prior gestational DM who were followed-up from 1991 to 2005. Adherence to the MD was assessed with the aMED score and a modified version of the aMED score ^†^.	Women in the highest quartile of adherence to the MD (mean aMED = 6.6 ± 0.7) exhibited a 40% (95% CI 0.44–0.82) reduced risk of T2DM, compared with those in the lowest quartile (mean aMED = 1.6 ± 0.6); the association was weakened after adjusting for BMI in the multivariate analysis (*p* = 0.13). Results were similar for the modified aMED score.
Abiemo *et al*. [24]	Prospective cohort study with a sample of 5390 initially free of T2DM individuals from the Multi-Ethnic Study of Atherosclerosis (MESA), who were followed up for a median of 6.6 years. Adherence to the MD was assessed with the aMD score ^#^.	Participants in the highest quintile of adherence to the MD (aMD score > 6) exhibited lower baseline glucose and insulin levels, compared with those in the lowest quintile (aMD score < 4); however longitudinal analysis revealed no significant association between adherence to the MD and risk of T2DM (HR = 1.02, 95% CI 0.95–1.10, *p* = 0.51).
Rossi *et al*. [25]	Prospective cohort study with a sample of 22,295 initially free of T2DM individuals from the Greek cohort of the European Prospective Investigation into Cancer and Nutrition (EPIC) study, who were followed for a median of 11.34 years. Adherence to the MD was assessed using the MDS score ^§^.	Participants in the highest quartile of adherence to the MD (MDS > 5) exhibited a 12% (95% CI 0.78–0.99) reduced risk of T2DM, compared with those in the lowest quartile (MDS < 4); in stratified analysis according to BMI values, adherence to the MD was inversely associated with T2DM risk only in overweight participants (HR = 0.87, 95% CI 0.77, 0.98).

MD: Mediterranean diet, (T2)DM: (type 2) diabetes mellitus, CVD: cardiovascular diseases, BMI: body mass index, HR: hazard ratio; * Index components (*n* = 5): consumption of raw vegetables, cooked vegetables, fruits, fish and olive oil; scoring system: 4 partitions for each component (0, 1, 2 or 3 points) based on absolute cut-off points; score range: 0–15; ^§^ Index components (*n* = 9): consumption of vegetables, legumes, fruits and nuts, grains, fish and seafood, meat and meat products, dairy products, and alcohol, as well as diet’s MUFA:SFA ratio; scoring system: 2 partitions for each component (0 or 1 points) based on the sex-specific median of components’ intake (for alcohol intake absolute cut-off points are used); score range: 0–9; ^†^ Index components (*n* = 9): consumption of vegetables, legumes, fruits, nuts, whole grains, fish, red and processed meat, and alcohol, as well as diet’s MUFA:SFA ratio; scoring system: 2 partitions for each component (0 or 1 points) based on the median of components’ intake (for alcohol intake absolute cut-off points are used); score range: 0–9; in the modified version of the aMED score, the MUFA:SFA component is omitted; ^‡^ Index components (*n* = 9): consumption of vegetables, legumes, fruits and nuts, grains, fish and seafood, olive oil, meat and meat products, dairy products, and alcohol; scoring system: 3 partitions for each component (0, 1 or 2 points) based on tertiles of components’ intake (for alcohol intake absolute cut-off points are used); score range: 0–18; ^#^ Index components (*n* = 10): consumption of vegetables, legumes, fruits, nuts, whole grains, fish, red and processed meat, whole-fat dairy products and alcohol, as well as diet’s MUFA:SFA ratio; scoring system: 2 partitions for each component (0 or 1 points) based on the median of components’ intake (for alcohol intake absolute cut-off points are used); score range: 0–10.

## 4. Mediterranean Diet and Treatment of Diabetes and Diabetes-Related Complications

Several dietary interventions for the treatment of DM have been explored during the last few decades. Given the fact that diabetic patients are at high cardiovascular risk, the cardio-protective low-fat diet has been traditionally used as part of the disease’s management. However, research has shown that there is no “one-size-fits-all” eating pattern for individuals with DM. According to the 2013 American Diabetes Association nutrition therapy recommendations for the management of adults with diabetes [27], both the macronutrient composition and the combinations of foods or food groups consumed in a diet should be based on individualized assessment of current eating patterns, personal preferences and metabolic goals. A Mediterranean-style dietary pattern is recommended as an effective alternative to a lower-fat, higher-carbohydrate eating pattern for T2DM patients due to emerging evidence of its beneficial effect on glycemic control and CVD risk factors (evidence rating: B, indicating supportive evidence from well-conducted cohort studies or case-control studies). Indeed, several epidemiological and interventional studies, described below, have demonstrated a beneficial effect of the MD on T2DM patients’ glycemic control, cardiovascular risk, as well as liver and sexual function.

### 4.1. Glycemic Control and Insulin Sensitivity

Evidence regarding the beneficial role of the MD on T2DM patients’ glycemic control is available through some cross-sectional studies, reporting better glucose homeostasis indices in diabetic patients closely adhering to the MD, compared with low adherers [28,29]; however, as mentioned previously, cross-sectional studies do not provide a good basis of causality and their results remain of limited value. More consistent and reliable are the results of RCTs evaluating the effect of the MD on T2DM patients’ glucose homeostasis indices [30,31,32,33,34,35,36], which are briefly presented in Table 2. Out of the seven RCTs, four were specifically designed to evaluate the effect of the MD on T2DM patients’ glycemic control [30,33,34,35], whereas in the other three, T2DM patients were a subgroup of the study sample [31,32,36]. Most of these studies demonstrate the beneficial effect of the MD on T2DM patient’s glycemic control and insulin sensitivity, as well as its superiority over control diets, *i.e*., a low-fat diet or usual dietary habits. This is also supported by the results of a recent review and meta-analysis by Ajala *et al*. [37] of twenty RCTs (overall 3073 diabetic patients) exploring the effect of different dietary approaches for DM treatment, also including three of the aforementioned RCTs [30,33,34]. According to the results of the meta-analysis, the low-carbohydrate, the low glycemic index, the high-protein and the Mediterranean diets all led to a greater improvement in glycemic control [HbA1c reductions of −0.12% (*p* = 0.04), −0.14% (*p* = 0.008), −0.28% (*p* < 0.001) and −0.41% (*p* < 0.001) respectively, compared with their respective control diets. Interestingly, the largest effect size was observed for the MD, when compared with usual care [30] or a low-fat diet [33,34].

**Table 2:** Interventional studies exploring the effect of the Mediterranean diet on type 2 diabetes mellitus patients’ glucose homeostasis indices.

Reference	Study Sample and Design	MD Intervention Details	Results
Toobert *et al*. [30]	6-month randomized controlled clinical trial: 279 postmenopausal women with T2DM were assigned to either a comprehensive lifestyle self-management program that also included a Mediterranean low-saturated fat diet (Mediterranean lifestyle program-MLP) or usual care.	Participants’ macronutrient intake was individualized. The MD recommended increased amounts of bread, vegetables, legumes and fish; less red meat, substituting poultry; no day without fruit; and avoidance of butter and cream, substituting olive and canola oils or margarines.	Subjects allocated to the MLP group exhibited lower HbA1c levels, compared with the control group (*p* < 0.001). However, the relative role of the MD in the context of the program that also included exercise, group support, smoking cessation and stress management training is unclear.
Estruch *et al*. [31]	3-month randomized controlled clinical trial: 772 individuals at high cardiovascular risk, including 421 T2DM patients, were assigned to one of three diets; a low-fat diet (LFD) based on the 2000 AHA guidelines, a MD supplemented with 1 L/week of extra-virgin olive oil or a MD supplemented with 30 g/day of mixed tree nuts.	Personalized dietary advice on the desired frequency of specific foods’ consumption was given to each participant, based on the assessment of their individual MD scores. Energy restriction was not recommended. Nutritional education was more intense for the participants assigned to the MD groups, compared with the LFD group.	Subjects allocated to the MD supplemented with olive oil and nuts groups exhibited lower glucose (*p* = 0.017 and *p* = 0.039, respectively), insulin (*p* = 0.001 and *p* < 0.001, respectively) and HOMA-IR levels (both *p* < 0.001), compared with subjects in the LFD group. No separate analysis for diabetic patients was performed.
Shai *et al*. [32]	2-year randomized controlled clinical trial: 322 moderately obese subjects, including 46 T2DM patients, were assigned to one of three diets; a calorie-restricted low-fat diet (CRLFD) based on the 2000 AHA guidelines, a calorie-restricted MD (CRMD), or a low-carbohydrate diet (LCD) based on the Atkins diet.	EI was restricted to 1500 kcal/day for women and 1800 kcal/day for men, with a goal of no more than 35% of EI from fat. The CRMD was rich in vegetables and low in red meat, with poultry and fish replacing beef and lamb. The main sources of added fat were 30 to 45 g/day of olive oil and a handful (<20 g/day) of nuts.	Among T2DM patients, only those in the CRMD group exhibited a decrease in glucose levels (*p* < 0.001 compared with the CRLFD group). Insulin and HbA1c levels decreased similarly in all three groups. The decrease in HOMA-IR was significantly greater in patients assigned to the CRMD compared with those assigned to the CRLFD (*p* = 0.04).
Esposito *et al*. [33]	4-year randomized controlled clinical trial: 215 overweight adults with newly-diagnosed T2DM who were not receiving anti-hyperglycemic drug therapy and had HbA1c levels <11% were assigned to either a low-carbohydrate MD (LCMD) or a low-fat diet (LFD) based on the 2000 AHA guidelines.	EI was restricted to 1500 kcal/day for women and 1800 kcal/day for men, with ≤50% and ≥30% of EI from carbohydrates and fat, respectively. The LCMD was rich in vegetables and whole grains and low in red meat, which was replaced with poultry and fish. The main source of added fat was olive oil (30–50 g/day).	At the end of the intervention 44% of patients in the LCMD group and 70% in the LFD group required treatment (*p* < 0.001). Improvements were greater in the LCMD group for glucose, HOMA-IR and HbA1c levels. The proportion of participants who met ADA goals for HbA1c was greater in the LCMD group.
Elhayany *et al*. [34]	12-month randomized controlled clinical trial: 259 overweight adults with T2DM were assigned to one of tree diets; a low-carbohydrate MD (LCMD), a traditional MD (TMD) or a low-fat diet (LFD) based on the 2003 ADA guidelines.	LCMD: 35% and 45% of EI from carbohydrates and fat, respectively. TMD: 50% and 30% of EI from carbohydrates and fat, respectively. Daily energy (20 kcal/kg), protein (20% of EI), fiber (30 g), sodium (≤3 g), potassium (>3 g), calcium (~1300 mg) and magnesium (>800 mg) intakes were similar in both diets.	Glucose, HOMA-IR and HbA1c decreased while insulin levels increased in all three groups. Changes in glucose, insulin and HOMA-IR levels were similar among groups. The reduction in HbA1c levels was significantly greater for patients allocated to the LCMD and TMD groups, compared with patients on the LFD (*p* = 0.021).
Itsiopoulos *et al*. [35]	Randomized cross-over interventional study: 27 T2DM subjects were assigned to either an *ad libitum* MD or their usual diet for 12 weeks and then cross over to the alternate diet.	Subjects in the MD group were provided with most of the meals and staple foods included in the traditional Cretan MD in excess of their energy requirements and advised to consume them *ad libitum*.	The *ad libitum* MD led to a significantly greater decrease in HbA1c, compared with the usual diet (*p* = 0.012). A similar trend for lower HOMA-IR values in the MG group was also observed (*p* = 0.061).
Lasa *et al*. [36]	12-month randomized controlled clinical trial: 191 T2DM subjects were assigned to one of three diets; a low-fat diet (LFD) based on the 2000 AHA guidelines, a MD supplemented with 1 L/week of extra-virgin olive oil or a MD supplemented with 30 g/day of mixed tree nuts.	Both MD groups received education on: abundant use of olive oil for cooking and dressing; increased consumption of fruits, vegetables, legumes and fish; reduction in total meat consumption; preparation of home-made sauce for dressing; avoidance of butter, cream, fast food, sweets, pastries and sugar-sweetened beverages; in alcohol drinkers moderate consumption of red wine.	HOMA-IR levels were not modified by dietary interventions (all *p* > 0.05). The adiponectin/leptin ratio increased in all three trial arms (all *p* < 0.05), while the adiponectin/HOMA-IR ratio increased in the MD supplemented with olive oil group (*p* = 0.027) and showed a trend towards increase in the MD supplemented with nuts (*p* = 0.069) and the LFD (*p* = 0.061) groups.

MD: Mediterranean diet; T2DM: type 2 diabetes mellitus; HbA1c: glycosylated hemoglobin; HOMA-IR: homeostasis model of assessment–insulin resistance; ADA: American Diabetes Association; AHA: American Heart Association; EI: energy intake; mg: milligram; g: gram; kg: kilogram; kcal: kilocalories.

### 4.2. Cardiovascular Risk and Related Mortality

Several RCTs [31,33,34,38] have demonstrated the beneficial effect of the MD on CVD risk factors (body mass index, waist circumference, blood lipids, blood pressure, inflammatory markers and adhesion molecules) in T2DM patients, as well as the advantage of the MD at improving these indices compared with a low-fat diet. However, data regarding the role of the MD in CVD prevention among T2DM patients are currently limited. So far, the association between adherence to the MD and likelihood of CVD in T2DM patients has been specifically explored only in one cross-sectional study. Briefly, in 2003, Ciccarone *et al*. [39] conducted a case-control analysis in a sample of 144 T2DM patients with peripheral arterial disease, matched for age and sex with 288 T2DM patients without macrovascular complications, from a total cohort of 944 Italian T2DM patients. In multivariate analysis, a higher MD score was associated with a 56% (95% CI 0.24–0.83) lower probability of peripheral arterial disease, independently of diabetes duration and presence of hypertension.

The effect of the MD on CVD associated mortality has been extensively studied over the past decades. The first study to provide strong data on the protective role of the MD against both fatal and non-fatal CVD complications was the Lyon Diet Heart Study, a randomized single-blind secondary prevention trial, comparing the effect of a Mediterranean-style alpha-linolenic rich diet and that of a prudent Western-type diet on CVD complication recurrence after a first myocardial infraction [40]. Although there are no available studies exploring the association between adherence to the MD and risk of developing CVD or CVD-associated mortality specifically in T2DM, there is one prospective cohort study [41] and two RCTs [42,43] that included a sufficient subgroup of diabetic patients. In a sample of 16,610 males and 23,860 females from the Melbourne Collaborative Cohort Study, of which 2150 had impaired glucose homeostasis, a small reduction in total and CVD-related mortality risk per 1-unit increase in the MD score used was observed (total mortality: Hazard ratio (HR) = 0.96, 95% CI 0.93–0.99 in men and HR = 0.94, 95% CI 0.92–0.97 in women; CVD mortality: HR = 0.94, 95% CI 0.89–0.99 in men and HR = 0.94, 95% CI 0.87–1.01 in women), over an average follow-up of 12.3 years [41]. Results of the GISSI-Prevenzione trial, including 11,323 survivors of myocardial infarction from Italy, of which 1700 were diabetic, revealed a 15% (95% CI 0.72–0.78) reduced risk of mortality per 1-unit increase in the MD score used over a mean of 6.5 years [42]. Finally, results of the PREDIMD study, in a sample of 7447 individuals at high cardiovascular risk, including 3614 T2DM subjects, revealed a 30% (95% CI 0.54–0.92) and a 28% (95% CI 0.54–0.96) reduced risk for cardiovascular events (myocardial infarction, stroke or death from cardiovascular causes) for participants assigned to a MD supplemented with olive oil and a MD supplemented with nuts, respectively, compared with the low-fat diet control group, after a median follow-up of 4.8 years [43].

### 4.3. Liver Function

It is estimated that up to 70% of T2DM patients suffer from non-alcoholic fatty liver disease, a pathological condition characterized by excessive hepatocyte triglyceride accumulation, resulting mainly from insulin resistance [44]. So far, one RCT has specifically evaluated the effect of the MD on liver function in patients with T2DM [45]. Briefly, 259 obese T2DM subjects were randomly assigned to a low-fat diet, a low glycemic index diet or a MD. At the end of the 12-month intervention, alanine aminotransferase levels decreased significantly in all three trial arms, compared with baseline (all *p* < 0.002), while patients in the MD group exhibited the lowest levels (*p* < 0.001 for the between-group difference); these differences were not mediated by weight loss, or improvements in insulin resistance index and triglyceride levels. The beneficial effect of the MD on liver function is also supported by a recent randomized cross-over interventional study, showing greater improvements in insulin sensitivity and hepatic steatosis in patients with biopsy-proven non-alcoholic fatty liver disease allocated to a MD, compared with those following a low-fat high-carbohydrate diet, in the absence of significant weight loss [46]. Clearly, more studies assessing liver function using imaging techniques or even liver biopsy data are required, in order to substantiate the effect of the MD on diabetic patients’ liver function.

### 4.4. Sexual Function

Sexual dysfunction has been recognized as a common complication in DM [47]. The association between adherence to the MD and sexual function in T2DM patients has only recently been explored. In 2010, Giugliano *et al*. [48] observed that the proportion of sexually active men significantly increased across tertiles of adherence to the MD (from 65% to 74%, *p* = 0.01) in a sample of 555 male T2DM patients; in addition, men with the highest MD score had a lower prevalence of both global (52% *vs*. 62%, *p* = 0.01) and severe erectile dysfunction (17% *vs*. 26%, *p* = 0.01), compared with low adherers. The same research team reported a significant increase in the proportion of sexually active women across tertiles of adherence to the MD (from 54% to 65%, *p* = 0.01) and a strong inverse association between adherence to the MD and the prevalence of sexual dysfunction (48%, 54%, and 58% in the upper, middle, and lower tertile of adherence, respectively, *p* = 0.01) among 595 female T2DM patients [49]. Given the cross-sectional nature of the above mentioned studies, the link between adherence to the MD and sexual dysfunction in diabetic patients remains to be evaluated in future prospective studies or clinical trials, for safe conclusions to be drawn.

## 5. Protective Mechanisms of the Mediterranean Diet in Diabetes

Several characteristics of the MD have been proposed to explain its beneficial effect on DM; however, the exact mechanisms remain partly elucidated. In 2007, Schroder [50] reviewed the association between the MD and T2DM development and proposed both indirect (via weight control) and direct (via consumption of foods rich in nutrients with various health benefits) effects of the MD against the disease.

It is well established that excessive body weight, particularly abdominal fat deposition, results in insulin resistance progression and is thus considered one of the major risk factors for T2DM development [51]. Consequently, diets preventing weight gain exert an indirect protective effect against the disease. The association between MD and anthropometric indices has been explored in several epidemiological studies and, although results remain controversial, there is much evidence to support an inverse association between adherence to the MD and likelihood of obesity [52]. In addition, according to the results of a recent meta-analysis by Esposito *et al*. [53], the MD was found to result in greater weight loss, compared with control diets (mostly a low-fat diet), especially when combined with energy restriction, physical activity or when adopted for more than six months. The aforementioned data, along with the fact that the MD is highly palatable and therefore well-tolerated among dieters [52], indicate its beneficial effect on maintaining a healthy body weight and therefore preventing T2DM development.

In addition to its beneficial effect on body weight, the unique combination of foods and nutrients found in the MD has been proposed to be beneficial for T2DM prevention and treatment. In particular, the high consumption of fruits, vegetables, legumes, nuts, whole-grain cereals and olive oil encouraged in the MD, leads to a high ratio of monounsaturated to saturated fatty acids, a low intake of trans fatty acids and a high intake of dietary fiber and antioxidants [50]. Regarding dietary fat, replacing saturated and trans with unsaturated (polyunsaturated and/or monounsaturated) fatty acids has been found to exert beneficial effects on insulin sensitivity and reduce the risk of T2DM development [54]. In addition, according to consistent epidemiological and clinical evidence, a high intake of dietary fiber (in particular cereal fiber) and antioxidants, as well as a high consumption of foods including these compounds, such as fruits, vegetables, legumes and whole grains, is associated with improved insulin sensitivity, improved pancreatic β-cell secretory capacity and reduced risk of T2DM development [55,56].

Besides the beneficial properties of individual components of the MD, the whole dietary pattern also presents strong anti-inflammatory and anti-oxidative effects. In specific, adherence to the MD has been consistently associated with decreased biomarkers of subclinical inflammation [57,58] and increased levels of adiponectin, both in healthy individuals [59] and T2DM patients [60]; data regarding adiponectin are of great importance, since increased levels of this metabolically active anti-inflammatory cytokine have been inversely associated with T2DM risk [61]. Moreover, the MD has been proposed to protect individuals from oxidative stress, defined as the persistent imbalance between free radical formation and antioxidant defense, which in turn seems to play a crucial role in the development of insulin resistance and beta cell dysfunction [56]. Indeed, adherence to the MD has been consistently associated with lower blood levels of oxidative molecules (e.g., oxidized low density lipoprotein particles, thiobarbituric acid reactive substances, 8-hydroxy-2-deoxyguanosine, *etc*.) and higher blood antioxidant capacity [62,63]. The aforementioned data support the potential protective role of the MD against pathological conditions, in which oxidative stress and inflammation are involved, including T2DM.

## 6. Conclusions

In the present review, the role of the MD in DM prevention and treatment, as well as its potential protective mechanisms against the disease, were briefly presented. According to epidemiological data, a greater adherence to the MD, as assessed by various MD indices, is inversely associated with T2DM risk in the general population, in individuals at high cardiovascular risk and in patients with established CVD. Interventional studies also demonstrate the beneficial role of the MD in T2DM management, with patients allocated to a MD exhibiting greater improvements in glycemic control and CVD risk factors, compared with those following a control diet. Interestingly, there is evidence that the MD may also have a beneficial role in the primary and secondary prevention of CVD and a favorable effect on liver and sexual function in diabetic patients, however sufficient data are currently lacking.

Two major points raised in the present review require consideration. Firstly, the use of “*a priori*” indices for the assessment of adherence to the MD in epidemiological studies is characterized by many methodological limitations. For instance, the wide variety of available MD scores makes it difficult to compare the results of studies, in which different scores are used; this observation, along with the fact that diet varies significantly across populations, suggest that an analysis of this type cannot provide universally applicable results [64]. In addition, MD indices may not precisely describe the overall MD, since most available scores focus on selected aspects of the diet and involve some level of arbitrary decision in the type and number of components to be included as well as their scoring system [64]. Secondly, the availability of few controlled trials designed to evaluate the metabolic and cardiovascular outcomes of the MD specifically in DM, as well as the fact that most clinical studies focused on surrogate markers for early CVD risk assessment, signify major limitations of the present review. Moreover, although some evidence regarding the association between adherence to the MD and risk of developing CVD or CVD-associated mortality is available from studies incorporating a subgroup of subjects with T2DM, this evidence is not specific to diabetic patients and should therefore be interpreted with caution.

In conclusion, although current data are insufficient, mounting evidence suggests the beneficial effect of the MD on T2DM prevention and treatment. Given the recent worldwide rise of the prevalence of the disease, as well as the various established health benefits of the MD, strategies aiming to promote adherence to this dietary pattern are of considerable public health interest.
